# Quality of Life and Functional Status in Individuals with Persistent Post-COVID Symptoms: A Cross-Sectional Comparison by Reported Rehabilitation

**DOI:** 10.3390/medicina61122214

**Published:** 2025-12-15

**Authors:** Michal Macej, Cyril Grus, Jakub Čuj, Lucia Demjanovič Kendrová, Wioletta Bronislawa Mikuľaková, Anna Kubincová, Peter Takáč

**Affiliations:** 1 Department of Physiotherapy, University of Prešov, 08001 Prešov, Slovakia; michal.macej@unipo.sk (M.M.); cyril.grus@unipo.sk (C.G.); jakub.cuj@unipo.sk (J.Č.); lucia.kendrova@unipo.sk (L.D.K.); wioletta.mikulakova@unipo.sk (W.B.M.); 2 Faculty of Medicine, Pavol Jozef Šafarik University, 04011 Košice, Slovakia; anna.kubincova@upjs.sk; 3 Department of Physical and Rehabilitation Medicine, L. Pasteur University Hospital, Rastislavova 43, 04001 Košice, Slovakia

**Keywords:** long COVID, rehabilitation, quality of life, functional status, SF-36, PCFS scale

## Abstract

*Background and Objectives*: Post-COVID-19 condition (PCC, long COVID) is associated with persistent symptoms and marked reductions in health-related quality of life (HRQoL), but real-world data on rehabilitation and everyday functioning remain limited. *Materials and Methods*: In a cross-sectional online survey conducted between 15 April and 15 May 2024, we analysed 406 adults (308 women; mean age 36.0 ± 12.1 years) with ongoing post-COVID symptoms recruited from two moderator-supervised support communities. The questionnaire included sociodemographic and clinical items, the 36-Item Short Form Health Survey (SF-36) and the Post-COVID-19 Functional Status (PCFS) scale. Participants indicated whether they had completed any form of rehabilitation targeting post-COVID problems (yes/no). Group differences were examined using Welch’s *t*-test, Mann–Whitney U and χ^2^ tests, as appropriate. Multiple linear regression models with Bonferroni correction were used to explore associations between rehabilitation status, age, sex, symptom duration and outcomes. *Results*: Overall, 182 respondents (44.8%) reported rehabilitation and 224 (55.2%) did not. The groups did not differ significantly in age, sex distribution, BMI, number of infections, symptom duration or hospitalisation history. Most SF-36 domains, component summaries and PCFS differed significantly between groups, with small-to-large effects favouring respondents who reported rehabilitation. The largest effect sizes were observed for Vitality and Mental Health, whereas Physical Functioning showed no clear difference. In multivariable models, older age and longer symptom duration were consistently associated with poorer HRQoL, while rehabilitation status remained a robust correlate of better scores in several SF-36 domains, both component summaries, perceived health, and lower PCFS grades after correction for multiple testing. *Conclusions*: Although the cross-sectional design, self-reported data and non-standardised rehabilitation exposure preclude causal inference, the findings highlight the substantial HRQoL and functional burden of long COVID and suggest that, within a symptomatic population, reported completion of rehabilitation is positively associated with multiple aspects of everyday health and functioning.

## 1. Introduction

Five years after the onset of the COVID-19 pandemic, a substantial proportion of individuals continue to report symptoms beyond the acute illness. Despite lower infection rates, post-COVID-19 condition (PCC, commonly known as long COVID) remains prevalent and clinically significant. Pooled data from 211 studies (>13 million participants) suggest that roughly 20–25% of adults report at least one symptom persisting for 12 weeks, with higher prevalence among previously hospitalised patients, women, and older adults [1].

PCC is heterogeneous. The dominant symptom clusters include fatigue, cognitive dysfunction, sleep disturbance, anxiety, and chronic pain. A 2023–2024 meta-analysis with two-year follow-up estimated fatigue at 28%, cognitive impairment at 27%, sleep problems at 21%, anxiety at 13%, and persistent pain at 8% [2]. Davis et al. estimated that the incidence of long COVID is around 10–15% in non-hospitalised cases, 50–70% in previously hospitalised patients and approximately 10–12% in vaccinated cases [3]. In the cross-national sample analysed by Bonsaksen et al., 18.4% of respondents reported COVID-19 infection [4]. Patients with long COVID often suffer from a wide range of symptoms, including fatigue, cognitive impairment, respiratory difficulties, cardiovascular problems, and psychological distress [5,6,7]. Establishing a causal relationship between a diagnosis of COVID-19 and subsequent morbidity is challenging, especially since chronic disease and persistent long COVID share risk factors such as older age, diabetes, smoking, malnutrition or obesity, immunosuppression, and hypertension [8].

These symptoms translate into meaningful losses in health-related quality of life (HRQoL). EQ-5D-5L studies consistently show lower utility values compared with matched controls (mean index ~0.93 vs. ~0.98), with patient self-ratings at or below levels seen in chronic heart failure and multiple sclerosis [9]. These symptoms have a significant negative impact on the functional status and quality of life of affected individuals [10,11].

Rehabilitation has emerged as a core management strategy. Interventions span structured exercise, respiratory training, psychological components, and hybrid or digital delivery. A living systematic review published in the BMJ of 24 randomized trials (*n* = 3695) concluded that combined physical-psychological rehabilitation or online Cognitive Behavioural Therapy (CBT) probably improves fatigue, concentration, and depressive symptoms (moderate certainty) [12]. With the increasing number of patients suffering from long COVID, rehabilitation has become an important part of the treatment plan, with clinical experience suggesting that physiotherapy and exercise programmes can improve physical fitness and muscle strength, leading to reduced fatigue and increased functional capacity of individuals [13,14,15,16,17,18]. Regarding the present strategies for long-term COVID rehabilitation, there are a number of unresolved issues that need to be addressed. The optimal approaches regarding the selection of rehabilitation methods, frequency, and duration of administration, as well as the assessment of outcomes, are still being explored. Further research is needed to establish evidence-based guidelines and standardise rehabilitation protocols to ensure the most effective support for this patient population [19]. General practitioners in several countries have also highlighted the need for the introduction of evidence-based standards of care [20]. It is also possible to assume that access to rehabilitation treatment varies across different regions and disparities can be observed depending on healthcare systems [21]. While comprehensive global statistics are limited, available data suggest that a relatively small percentage of long COVID patients receive such interventions.

More recent trials report clinically relevant gains: an eight-session, behaviourally informed outpatient programme (*n* = 314) produced a sustained ~9-point improvement on the SF-36 Physical Functioning subscale [22], and a fully online multimodal programme improved overall HRQoL at three months [23]. Yet access lags behind need. German claims data illustrate sharp growth in physiotherapy prescriptions (218 in 2020 to 6928 in 2022) [24], but workforce and capacity constraints persist. In 2023, only 1.8% of seven million workers received any certification related to PCC, and a WHO multi-country survey in 2024–2025 reported ongoing waiting-list growth and staffing shortages in rehabilitation services [25]. While the WHO definition of post-COVID-19 condition typically requires symptoms persisting for at least 12 weeks, our survey recruited adults with ongoing symptoms lasting ≥4 weeks, and we report symptom-duration strata explicitly to acknowledge that a minority of participants were in an earlier, post-acute phase.

Recent syntheses also indicate that respiratory physiotherapy—particularly high-intensity pulmonary rehabilitation aligned with American Thoracic Society/European Respiratory Society (American Thoracic Society/European Respiratory Society ATS/ERS) principles—can reduce long-COVID dyspnoea, but trial content and dosing remain variable, limiting comparability and precision of recommendations [18].

### 1.1. Evidence Gaps

Despite the rapid growth of the PCC literature, several gaps remain clinically important.

(1) Efficacy–effectiveness divide. Randomised trials and living reviews show that protocolised, CBT-informed multimodal rehabilitation probably reduces fatigue and cognitive symptoms, improves mood, and can raise self-reported physical function. However, these effects arise under tightly controlled conditions and do not mirror the heterogeneous rehabilitation delivered in routine care, where content, dose, setting, delivery mode, and adherence vary widely. External validity to patient-led and service-level pathways remains uncertain [12].
(2) Heterogeneity and incomplete specification of exposure. Observational reports combine diverse ingredients (aerobic/resistance training, breathing exercises, pacing/PEM-aware strategies, psychological elements) with variable intensity, duration, and delivery (in-person, hybrid, digital). Dose is inconsistently reported, and documentation of treatment actually received is often incomplete. The field still lacks core descriptors for rehabilitation components and harmonised outcome sets spanning SF-36 domains and functional scales such as the Post-COVID-19 Functional Status (PCFS) scale, which impedes synthesis and implementation [12].
(3) Access and system constraints. Even where benefit is plausible, uptake is limited by capacity, workforce pressures, long waiting lists, and fragmented single-specialty models that struggle to deliver interdisciplinary, symptom-paced care at scale. These pressures have persisted beyond the acute pandemic period [26].

These gaps have consequences. Large population cohorts show substantial decrements in HRQoL on generic instruments (EQ-5D-5L, SF-36), with quantifiable utility losses now documented in big-data resources (e.g., Open-PROMPT/OpenSAFELY), underscoring the need for scalable, real-world solutions [27].

In the context of post-COVID-19 condition, rehabilitation is conceptualised as a set of structured interventions delivered or supervised by health professionals (e.g., physiotherapists, physicians, occupational therapists, psychologists) to address limitations in physical, cognitive and psychosocial functioning. These may include supervised exercise or activity management, respiratory and musculoskeletal physiotherapy, education about pacing and energy conservation, cognitive strategies to address attention and memory problems, and psychological or behavioural interventions targeting anxiety, low mood and adjustment difficulties. In routine clinical practice, such programmes are heterogeneous in content, format (inpatient, outpatient or community-based) and intensity, and may be complemented by elements of home-based self-management.

### 1.2. Aim of the Study

In light of these gaps between protocolised clinical trials and heterogeneous real-world rehabilitation, the present study aimed to address three questions. First, we sought to describe health-related quality of life and functional status, as measured by SF-36 domain and summary scores and the Post-COVID Functional Status (PCFS) scale, in a cross-sectional sample of adults with post-COVID-19 condition recruited from two national online support communities. Second, we examined whether self-reported completion of rehabilitation was associated with differences in these outcomes. Third, we explored which patient factors (including age, sex, symptom duration, comorbidities and other covariates) were associated with health-related quality of life and functional status in multivariable models. Given the cross-sectional design and the non-standardised nature of rehabilitation programmes, these analyses were intended to explore associations and generate hypotheses for future prospective evaluations, rather than to infer causal treatment effects.

## 2. Materials and Methods

A total of 406 respondents (308 women and 98 men) with persistent symptoms following COVID-19 participated in this survey-based observational study, forming a symptomatic post-COVID-19 sample with predominantly long-lasting symptoms. The mean age was 36.0 ± 12.1 years. Their participation in this study was voluntary.

### 2.1. Sample and Recruitment

Recruitment was conducted between 15 April and 15 May 2024, via two private, moderator-supervised Facebook support communities for individuals with ongoing post-COVID-19 symptoms—“long COVID a postcovidový syndrom—ČESKO A SLOVENSKO” (≈4500 members) and “COVID následné potíže” (≈2700 members)—where access requires prospective members to confirm a history of SARS-CoV-2 infection and persistent health complaints. An anonymous online survey was posted as a pinned announcement in each group. From a combined membership of approximately 7200, a total of 406 individuals (308 women, 98 men) completed the survey (≈5.6% response). Participants were recruited exclusively from these two Facebook support communities for individuals with ongoing post-COVID-19 complications. No additional recruitment channels (e.g., hospital clinics, primary care practices) were used, and the resulting sample is therefore best described as a non-probability convenience sample of members of these online support communities.

In addition to general data (gender, age, weight, and height), patients completed the Patient Self-Report Methods for the Post-COVID-19 Functional Status Scale (PCFS) and a structured Quality of Life questionnaire (SF-36). The respondents also answered the question of whether they had completed any form of rehabilitation, which was the key information used to divide the obtained questionnaires into two groups: those that had completed rehabilitation (*n* = 182) and those that had not (*n* = 224).

The inclusion criteria were men and women aged ≥18 years with persistent symptoms following COVID-19, lasting at least four weeks after recovery from the last episode of COVID-19. This long-lasting threshold captures individuals with post-acute and longer-term post-COVID-19 symptoms. As shown in Table 1, the majority of respondents reported symptom duration of ≥4 months, but approximately one-fifth had symptoms lasting 1–3 months at the time of the survey. The persistence of symptoms was assessed using specific questions in the questionnaire in the appendix. The full versions of all study-specific questionnaire items used in this study are provided in Supplementary File S1. Standardised instruments (SF-36 and the Post-COVID Functional Status Scale) are not reproduced in full due to copyright restrictions.

As shown in Table 1, over 80% of respondents reported persistent symptoms for at least four months (4–6, 7–12 or >12 months), whereas around 20% were in the 1–3-month range at the time of the survey. The sample therefore represents a symptomatic post-COVID-19 group with predominantly long-lasting symptoms rather than an exclusively ≥12-week ‘long-COVID’ population.

The exclusion criteria included recovery from COVID-19 without any persistent or long-term respiratory, cardiovascular, neurological, haematological, psychological, or infectious symptoms associated with the disease; presence of difficulties listed in the questionnaire that were already experienced before contracting COVID-19; and lack of willingness to cooperate or refusal to consent to participate in the research study. The study protocol was approved by the Ethics Commission of the University of Prešov in Prešov under the number 04/2023.

Participants were asked whether they had completed any rehabilitation programme specifically intended to address their post-COVID-19 symptoms, broadly defined to include physiotherapy-based programmes, multidisciplinary rehabilitation, or other structured interventions prescribed or supervised by health professionals. For the purposes of the present analysis, rehabilitation was coded as a binary variable (completion of any such programme: yes/no), and we did not standardise or stratify by specific modality, setting or intensity.

### 2.2. Measures

#### 2.2.1. Patient Self-Report Methods for the Post-COVID-19 Functional Status Scale (PCFS Scale)

This PCFS scale is used for tracking functional status over time as well as for research purposes [28]. It covers the full spectrum of functional outcomes and focuses on both limitations in usual duties/activities and changes in lifestyle in five scale grades. The tool incorporates limitations in daily activities as well as lifestyle changes. PCFS Grades represent an increasing range of functional impact, from Grade 0 (no functional limitations), Grade 1 (negligible functional limitations), Grade 2 (slight functional limitations), Grade 3 (moderate functional limitations), and Grade 4 (severe functional limitations)**.**

#### 2.2.2. Quality of Life Questionnaire (SF-36)

The 36-item Short Form Health Survey (SF-36) is an instrument for evaluating health-related quality of life. It assesses eight domains: Physical Functioning (PF), Role Limitations due to Physical Health Problems (Role-Physical, RP), Bodily Pain (BP), General Health (GH), Vitality (VT; energy/fatigue), Social Functioning (SF), Role Limitations due to Emotional Problems (Role-Emotional, RE), and Mental Health (MH; emotional well-being). All domain scores range from 0 to 100, with higher scores indicating better health. In addition, we calculated the standard Physical Component Summary (PCS) and Mental Component Summary (MCS) scores and extracted the single self-rated health item (“In general, would you say your health is…?”), referred to as Perceived Health Status. This single-item score is not part of the official SF-36 scoring and should be interpreted with caution; it does not replace PCS/MCS or the individual scales. Health-related quality of life was assessed using the 36-Item Short Form Health Survey (SF-36). For Czech respondents, the validated Czech version of the SF-36 was used. For Slovak respondents, we used a linguistically adapted version based on the Czech translation, with minor wording adjustments to reflect Slovak language usage, following standard practice for questionnaire adaptation in our setting [29,30].

All study-specific items used in the online questionnaire (demographic and clinical information, long COVID-related questions and questions on rehabilitation) are available in Supplementary File S1. Standardised instruments SF-36 and the Post-COVID Functional Status Scale (PCFS) are not reproduced in the Supplementary Materials because of copyright restrictions and are referenced in the main text instead.

### 2.3. Statistical Analysis

Continuous outcomes (SF-36 domain scores and PCS/MCS) were summarised as mean ± SD or median (IQR), and categorical variables as counts (%). Age was compared between groups using Welch’s *t*-test. Other continuous or ordinal outcomes were compared using the Mann–Whitney U test. Where applicable, categorical comparisons used the χ^2^ test (or Fisher’s exact test when expected counts were small). To control family-wise error, Bonferroni correction was applied in two contexts. For between-group comparisons of SF-36 domains and component summaries, raw *p*-values were adjusted within the family of eight SF-36 domains, with PCS and MCS considered separately. For the multivariable regression analyses, we controlled the family-wise error rate using a global Bonferroni correction across 37 prespecified predictor–outcome tests. For each predictor–outcome pair we calculated a Bonferroni-adjusted *p*-value (*p*_adj = min [*p* × 37, 1.00]; two-sided) and reported it alongside the corresponding standardised β coefficient in a separate table. Effect sizes are presented as rank-biserial correlation for Mann–Whitney tests and standardised β coefficients for multivariable models. For Mann–Whitney tests, effect size was expressed as the rank-biserial correlation r, oriented in a benefit-directed manner such that r > 0 indicates better outcomes in the rehabilitation group (including lower PCFS grades).

Multiple linear regression was used to examine associations between covariates (age, sex, symptom duration, comorbidities, prior COVID-19 episodes, history of hospitalisation, BMI, physical workload, physical activity, rehabilitation status) and SF-36 outcomes (domains and PCS/MCS). Two-sided tests with α = 0.05 were applied. Available-case analyses were performed (sample sizes per analysis reported in the Results). Analyses were conducted in JASP, version 0.19.3.

### 2.4. Sample Size Estimation

Given the absence of robust prior data on the prevalence of persistent post-COVID-19 symptoms in our target communities, we used a standard single-proportion formula with finite-population correction as a rough benchmark for the number of respondents required to estimate proportions with reasonable precision. Assuming a 95% confidence level (Z = 1.96), a 5% margin of error (e = 0.05), an expected proportion *p* = 0.50 (conservative), and using the Slovak resident population (*n* = 5,424,687) as a reference, the uncorrected size was n_0_ = 384.16 and the corrected size *n* ≈ 384. We therefore aimed to recruit ≥385 respondents. However, recruitment was conducted via two online support groups and thus represents a non-probability convenience sample, including both Slovak and Czech residents. The sample size calculation should therefore be interpreted as illustrative rather than as defining a formal sampling frame or inferential precision for the general population. Calculations were verified with DataStatPro, version 1.1.7 Beta (DataStatPro Team, 2024; accessed May 2024).

### 2.5. Most Common Persistent Symptoms

**Figure 1:**
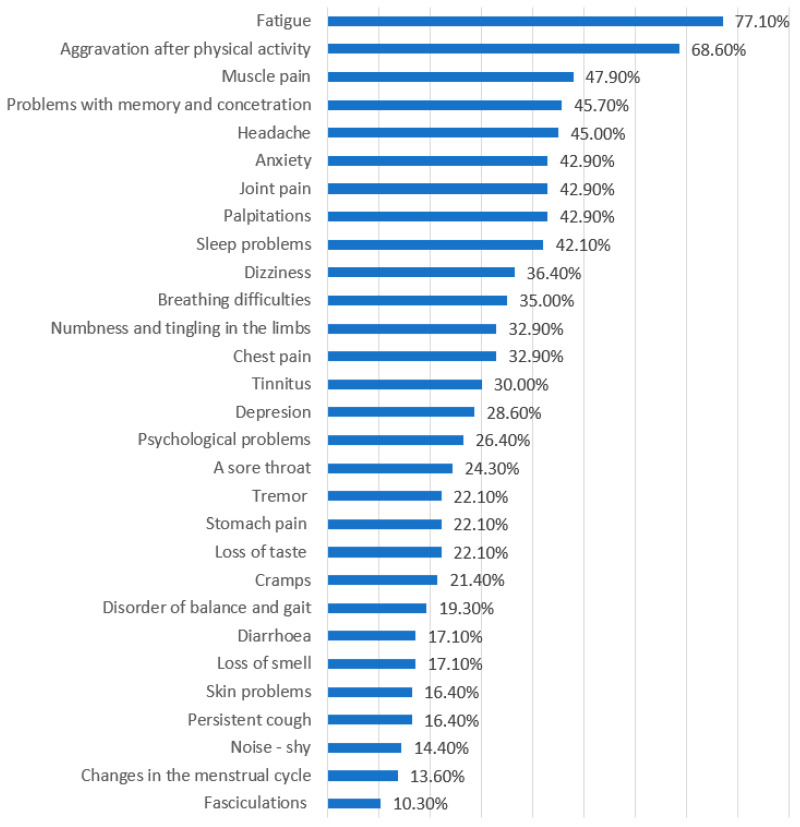
Most frequently reported persistent symptoms among respondents with post-COVID-19 condition (*n* = 406). Bars show the percentage of participants reporting each symptom; respondents could select more than one symptom.

The most commonly reported symptoms included fatigue (77.1%), muscle pain (68.6%), headache (47.9%), sleep disturbances (42.9%) and joint pain (42.9%). Respondents could indicate more than one symptom; less frequent complaints included hypersensitivity to sound, visual disturbances and so-called “COVID fingers” (Figure 1).

## 3. Results

We also conducted a homogeneity test to compare the group of patients who underwent rehabilitation with those who did not in terms of age, gender, and symptom duration.

No significant differences were observed between respondents who completed rehabilitation and those who did not with respect to age, sex distribution, symptom duration, number of prior COVID-19 infections, BMI, height, weight or hospitalisation history (all *p* > 0.05; Table 2). Although the proportion of previously hospitalised patients was numerically higher in the rehabilitation group (11.0% vs. 6.2%), the χ^2^ test (χ^2^ (1) = 2.94, *p* ≈ 0.086) did not indicate a statistically significant difference at the 5% level. These findings suggest that participation in rehabilitation was not strongly driven by the measured baseline characteristics.

### 3.1. HRQoL and Functional Status Differ Between Respondents with and Without Rehabilitation

In the next step (Table 3), we compared quality-of-life and functional outcomes between respondents who reported rehabilitation and those who did not.

Effect size is the rank-biserial correlation (r) reported in a benefit-oriented direction: r > 0 indicates better outcomes in the rehabilitation group. For SF-36 domains (higher = better), positive r corresponds to higher scores in the rehabilitation group. For PCFS (higher = worse), we reversed the direction so that positive r corresponds to lower PCFS (less limitation) in the rehabilitation group. Magnitude: ~0.10 small, ~0.30 medium, ≥0.50 large. All *p*-values shown are unadjusted (raw, two-tailed); family-wise error for SF-36 domains and component summaries was controlled using Bonferroni correction as described in Section 2.3.

All indicators except Physical Functioning differed significantly between groups (*p* < 0.001). Effects favoured the rehabilitation group, with the largest differences for Vitality (r = 0.743) and Mental Health (r = 0.572), whereas Physical Functioning showed only a borderline difference (*p* = 0.052). Positive r denotes better outcomes in the rehabilitation group for all measures, including lower functional limitation on PCFS.

**Figure 2:**
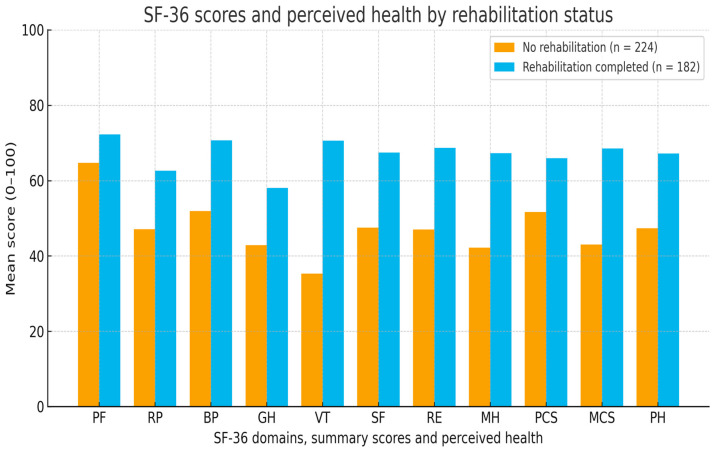
SF-36 health-related quality of life scores and perceived health by rehabilitation status (*n* = 406; no rehabilitation, *n* = 224; rehabilitation, *n* = 182). Higher scores indicate better health-related quality of life/perceived health. Abbreviations: PF, Physical Functioning; RP, Role Limitations due to Physical Health; BP, Bodily Pain; GH, General Health; VT, Vitality; SF, Social Functioning; RE, Role Limitations due to Emotional Problems; MH, Mental Health; PCS, Physical Component Summary; MCS, Mental Component Summary; PH, Perceived Health (SF-36 single-item perceived health status).

These between-group differences are illustrated in Figure 2, which shows consistently higher mean SF-36 domain and summary scores, as well as better perceived health, in respondents who completed rehabilitation compared with those who did not. The largest gaps are visible for Vitality, Mental Health and the Mental Component Summary, indicating clinically meaningful advantages in energy, mood and psychosocial functioning among participants who reported rehabilitation.

### 3.2. Predictors of HRQoL and Functional Status in Multivariable Models

#### 3.2.1. Analysis of Predictors Influencing Health Indicators

We investigated whether potential predictors were associated with individual domains of quality of life and functional status. Multiple linear regression models were fitted in the full sample (*n* = 406), using the following predictors: sex, age, BMI, physically demanding job, previous physical activity, number of past COVID-19 infections, duration of symptoms, hospitalisation, and completion of rehabilitation. The dependent variables were the SF-36 domain scores, PCS and MCS, and the PCFS score. Separate models were estimated for each outcome.

#### 3.2.2. Variable Coding and Scaling

Variable coding. Categorical predictors were coded for the linear regression models as follows:

Gender: Female = 1, Male = 0 (reference).

Rehabilitation (completion of any rehabilitation programme): Yes = 1, No = 0 (reference).

Hospitalisation for COVID-19: Yes = 1, No = 0 (reference).

Physically demanding work: Yes = 1, No = 0 (reference).

Number of prior COVID-19 infections: entered as a non-negative integer (1, 2, 3, ≥4 recorded as 4).

Symptom duration (ordered categories 1–4, reflecting 1–3, 4–6, 7–12 and >12 months, respectively).

#### 3.2.3. Continuous Variables

Age (years), BMI (kg/m^2^), and SF-36 domain scores including PCS and MCS were analysed on their native continuous scales (SF-36: 0–100; higher = better). The PCFS grade (0–4; higher = worse) was modelled as an outcome in a separate analysis and treated as approximately continuous.

#### 3.2.4. Standardisation and Interpretation

We report standardised beta coefficients (β). With the above coding, a positive β for Rehabilitation indicates higher SF-36 scores (better health), while a negative β for Rehabilitation indicates a lower PCFS grade (less limitation). For Gender (Female = 1, Male = 0), a negative β means females scored lower than males on the given SF-36 outcome, all else equal. Because symptom-duration codes increase with longer duration, negative β values indicate worse outcomes with longer duration. Collinearity diagnostics (tolerance, VIF) did not indicate multicollinearity problems.

Standardised β coefficients are shown only for predictors that were statistically significant at *p* < 0.05 in the multivariable models. Predictors: Age (years), Sex (reference = male), Number of past COVID-19 infections, Symptom duration (months), Physically demanding job (yes/no), Rehabilitation (completion of a post-COVID rehabilitation programme: yes/no).

“Rehabilitation” in the presented model represents the participation of patients in the rehabilitation treatment.

The table presents the Beta Coefficients and *p*-values of significance in the same order as the predictors.

The collinearity statistics were assessed for all models using Tolerance and Variance Inflation Factor (VIF) values. A Tolerance value below 0.1 or a VIF above 10 was typically considered indicative of problematic multicollinearity.

Across all models, the Tolerance values ranged from 0.682 to 0.983, and the VIF values remained between 1.017 and 1.466; therefore, we did not assume the presence of substantial collinearity issues.

Multiple linear regressions identified several significant predictors of HRQoL and functional status (Table 4). In models using unadjusted *p*-values, completion of rehabilitation was positively associated with most SF-36 domains and both component summaries, with the largest standardised coefficients observed for Vitality (β = 0.645; *p* < 0.001) and Perceived Health Status (β = 0.406; *p* < 0.001). Age and longer symptom duration were consistently associated with poorer outcomes across several domains, whereas sex, number of past COVID-19 infections, physically demanding work and hospitalisation showed smaller or more selective associations.

### 3.3. Rehabilitation Status Is Positively Associated with Multiple HRQoL Domains and Lower PCFS Grades

Participants who reported completing rehabilitation differed from those who did not across multiple health-related parameters. In multivariable linear regression, completion of rehabilitation was positively associated with Vitality (β = 0.645, *p* < 0.001) and the Mental Component Summary (β = 0.475, *p* < 0.001). Additional positive associations were observed for General Health (β = 0.285, *p* < 0.001), Mental Health (β = 0.486, *p* < 0.001) and Perceived Health Status (β = 0.406, *p* < 0.001). Completion of rehabilitation was also positively associated with the Physical Component Summary (β = 0.285, *p* < 0.001) and with Bodily Pain (β = 0.274, *p* < 0.001). Significant associations were likewise noted for Role Limitations due to Physical Health (β = 0.202, *p* < 0.001), Social Functioning (β = 0.302, *p* < 0.001) and Role Limitations due to Emotional Problems (β = 0.261, *p* < 0.001). Finally, rehabilitation status was inversely associated with Post-COVID Functional Status (PCFS) scores (β = −0.246, *p* < 0.001), consistent with lower functional limitation among those who completed rehabilitation. Because multiple linear regression analyses were conducted, a Bonferroni correction was applied to account for multiple comparisons and to minimise the risk of Type I error, yielding a more conservative interpretation of statistical significance.

Bonferroni-adjusted *p*-values were computed using a global correction across 37 prespecified predictor–outcome tests (*p*_adj = min [*p* × 37, 1.00]; two-sided tests, coefficient degrees of freedom ≈ 396). For each predictor–outcome pair, Table 5 reports the corresponding standardised regression coefficient, raw *p*-value and Bonferroni-adjusted *p*-value. Where raw *p*-values were extremely small, we either report the numerical value (to four decimal places) or indicate *p* < 0.001; adjusted *p*-values were obtained by multiplying the raw *p* by 37 and truncating at 1.00. Associations with *p*_adj < 0.05 were considered statistically significant after correction for multiple testing.

After adjustment for multiple comparisons, completion of rehabilitation remained significantly associated with better scores in several SF-36 domains, including Role Limitations due to Physical Health, Bodily Pain, General Health, Vitality, Social Functioning, Role Limitations due to Emotional Problems and the Mental Health domain, as well as with higher Physical and Mental Component Summary scores, more favourable Perceived Health Status and lower PCFS grades (all *p*_adj < 0.05). In contrast, the association between rehabilitation and Physical Functioning did not reach the adjusted significance threshold (*p*_adj = 0.074). Age and longer symptom duration also remained significant negative predictors for multiple physical and mental health outcomes (for example, both were associated with lower General Health, Social Functioning, PCS, MCS and Perceived Health), whereas sex remained significantly associated only with Physical Functioning and the Physical Component Summary, and the number of past COVID-19 infections did not retain statistical significance for any outcome after Bonferroni correction.

After Bonferroni correction for multiple testing, the association between rehabilitation and Physical Functioning did not meet the adjusted significance threshold, whereas the associations with several other domains, both component summaries, perceived health and PCFS remained statistically significant.

**Figure 3:**
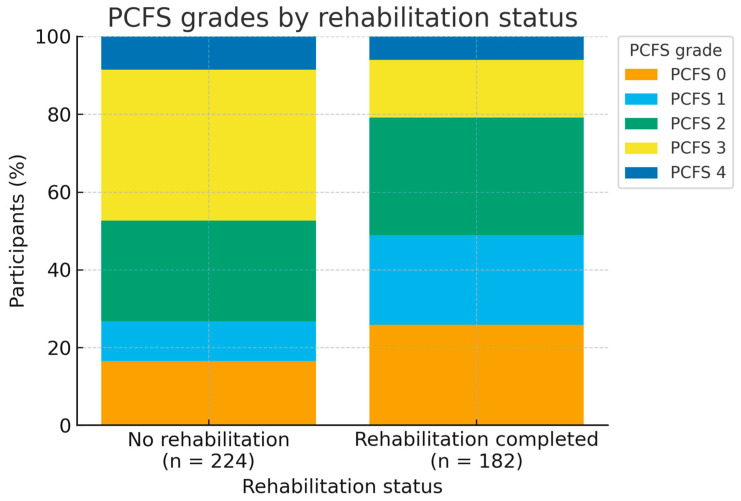
Distribution of Post-COVID Functional Status (PCFS) grades by rehabilitation status. (PCFS) grades by rehabilitation status (*n* = 406; no rehabilitation, *n* = 224; rehabilitation, *n* = 182). Higher PCFS grades indicate greater functional limitation.

Figure 3 illustrates the distribution of PCFS grades by rehabilitation status. Participants who completed rehabilitation were more likely to report mild or no functional limitations (PCFS 0–1), whereas moderate to severe limitations (PCFS 3–4) were more frequent among those who did not complete rehabilitation, underscoring the clinical relevance of the observed differences in functional status.

Effect sizes were directionally consistent and clinically coherent: negative associations for age and symptom duration were modest (β in the approximate range −0.16 to −0.22), indicating somewhat poorer health status among older participants and those with more prolonged symptoms. In contrast, rehabilitation showed some of the largest positive associations (for example, β ≈ 0.65 for Vitality and β ≈ 0.30 for Social Functioning), indicating better patient-reported health among respondents who reported having completed rehabilitation. These findings should, however, be interpreted within the constraints of the cross-sectional design and the potential for residual confounding, and are best viewed as hypothesis-generating rather than as definitive evidence of causal treatment effects.

## 4. Discussion

The need to optimise rehabilitation protocols for long COVID continues to be highlighted as health systems move beyond the acute phase of the pandemic [31]. Because long COVID is a multisystem condition, interventions must target a broad range of physical, cognitive and psychosocial consequences rather than focusing on a single organ system [31]. In this cross-sectional, survey-based study, we investigated a symptomatic post-COVID-19 sample of adults with predominantly long-lasting symptoms, comparing health-related quality of life and functional status between respondents who reported having completed rehabilitation and those who had not, and examining patient characteristics associated with these outcomes. Respondents who reported rehabilitation generally had more favourable SF-36 profiles and lower functional limitation on the PCFS, and younger age and shorter symptom duration were associated with better outcomes across several domains. However, because exposure and outcomes were assessed at a single time point, these findings reflect associations rather than causal effects of rehabilitation.

### 4.1. Group Comparison by Rehabilitation Status

Respondents who had completed rehabilitation reported higher scores in most SF-36 domains and on both component summaries, with the largest differences between groups observed for Vitality and Mental Health, whereas Physical Functioning showed only a borderline difference. Functional status on the PCFS scale was also more favourable in the rehabilitation group. These patterns are broadly consistent with earlier work demonstrating that long COVID is associated with substantial impairments across physical, psychological and social dimensions of health [9,10,11,32]. In a regional study, Líška et al. reported that patients with long COVID had significantly lower SF-36 scores than healthy controls across all domains, with particularly marked impairments in physical functioning and role limitations due to physical health [32]. Our between-group comparisons by rehabilitation status extend this picture by suggesting that, within a symptomatic long-COVID population, there is measurable heterogeneity in HRQoL and functional limitation that co-varies with reported rehabilitation exposure.

The magnitude and pattern of the differences observed in our study are compatible with findings from structured rehabilitation programmes. Multidisciplinary programmes combining supervised exercise, education and psychological support have been associated with improvements in physical performance, dyspnoea, fatigue and mental health in cohorts of patients post-COVID-19 (with long COVID) [15,16,17,18,33]. Recent trials of brief, protocolised interventions, including a behaviourally informed outpatient programme and an online multimodal programme, have also reported clinically relevant gains in SF-36 Physical Functioning and overall HRQoL [22]. Unlike these interventional studies, however, our analysis is observational and relies on self-reported rehabilitation of heterogeneous content and intensity. The differences between groups may therefore reflect a combination of rehabilitation effects, selection into rehabilitation, baseline severity, health-seeking behaviour and other unmeasured contextual factors.

### 4.2. Fatigue, Vitality and Mental Health

The particularly pronounced differences in Vitality and Mental Health are noteworthy given the central role of fatigue and psychological distress in long COVID. Fatigue is one of the most frequent and persistent symptoms reported after COVID-19, with high prevalence in both hospitalised and non-hospitalised cohorts [34,35]. Longitudinal research has identified fatigue as a key predictor of overall HRQoL, persistent symptoms and perceived stress one year after severe COVID-19 [36]. In this context, our observation that vitality and mental health scores were higher in the rehabilitation group is consistent with the broader literature and with interventional studies suggesting that multidisciplinary or psychologically informed rehabilitation can alleviate fatigue, mood symptoms and cognitive complaints [12,33,37].

The absence of a clearly significant difference in Physical Functioning warrants cautious interpretation. It may indicate that perceived improvements in vitality and mental health emerge even when limitations in physical activities persist, that changes in physical capacity lag behind psychological adaptation, or that the study was underpowered to detect more modest between-group differences in this domain. It is also possible that the SF-36 Physical Functioning scale is less sensitive than targeted performance-based measures for capturing changes relevant to long-COVID rehabilitation at the time point studied [7,32].

In multivariable models, older age and longer symptom duration were consistently associated with lower scores across several SF-36 domains and component summaries, and with less favourable perceived health. These associations align with large cohort and registry studies showing that older adults and those with more prolonged symptom trajectories tend to report lower HRQoL and greater functional impact after COVID-19 [7,9,27,35]. They are also in line with meta-analytic evidence that a proportion of patients experience symptoms persisting beyond two years, with fatigue, pain and cognitive problems contributing to long-term burden [8].

Rehabilitation status emerged as a robust correlate of better outcomes in several domains, even after adjustment for age, sex and symptom duration. Positive associations with Vitality, Social Functioning, Bodily Pain, General Health, Perceived Health Status and lower PCFS scores remained statistically significant after stringent Bonferroni correction, whereas the association between rehabilitation and the Physical Component Summary did not meet the adjusted significance threshold. These patterns are directionally consistent with the benefits reported in structured rehabilitation cohorts and trials [15,16,18,33,38].

From a mental health perspective, participants who completed rehabilitation reported better scores in several relevant SF-36 domains, including Vitality, Social Functioning and the Mental Health domain, and these domain-level differences remained statistically significant after Bonferroni correction. In line with this pattern, the Mental Component Summary (MCS) also showed a substantial association with rehabilitation (β ≈ 0.48), and this association persisted after applying the conservative Bonferroni adjustment (*p*_adj < 0.001). Taken together, these findings suggest that rehabilitation may be linked not only to improved physical functioning and symptom burden but also to better perceived mental health and psychosocial adjustment. However, because of the cross-sectional design and the possibility of residual confounding, these results should be interpreted as hypothesis-generating rather than as definitive evidence of causal treatment effects.

Several variables that might be expected to influence outcomes—such as BMI and history of hospitalisation—were not consistently associated with SF-36 domains or PCFS in our models. This may be due to limited variability in these measures within our sample, the relatively small number of previously hospitalised respondents, or the limitations of self-reported anthropometry in an online survey. Prior COVID-19 infections and physically demanding work showed only selective associations, underscoring the multifactorial nature of long-COVID sequelae and the need for more detailed characterisation of comorbidities, occupational demands and psychosocial context [4,19,37].

### 4.3. Rehabilitation Exposure, Heterogeneity and System Constraints

In our analysis, rehabilitation was treated as a binary, self-reported exposure [12,13,18]. Incomplete specification of “real-world” rehabilitation content, dose and fidelity across studies remains a major challenge for evidence synthesis and guideline development [12,37].

Our findings also speak to concerns about access and system-level constraints. Only a minority of long-COVID patients in many settings appear to receive structured rehabilitation, despite growing evidence that such programmes can improve symptoms and functional outcomes [15,16,21,24]. Workforce shortages, long waiting times and fragmented service configurations have been reported in several countries, including within Europe [20,26]. A recent WHO multi-country survey among rehabilitation workers in five European countries documented widespread disruption and suspension of services and increased patient waiting lists, indicating ongoing pressure on rehabilitation capacity beyond the acute phase of the pandemic [25]. Against this backdrop, our observation that respondents who reported rehabilitation tended to have better HRQoL and functional scores—while not implying causality—supports calls to systematically integrate multidisciplinary rehabilitation into long-COVID care pathways and to address inequities in access [19,31].

Several plausible mechanisms may underpin the observed associations between rehabilitation and better outcomes, although our cross-sectional design does not permit causal inference. First, structured rehabilitation programmes typically provide education about pacing, energy conservation and symptom-contingent activity planning, which may help patients avoid cycles of overexertion and post-exertional symptom exacerbation. Second, supervised physical and respiratory training can address deconditioning, dyspnoea and musculoskeletal pain, thereby improving physical functioning and fatigue perception. Third, psychological and behavioural components, including cognitive-behavioural or supportive interventions, may alleviate anxiety, low mood and health-related worry, which are known to interact bidirectionally with physical symptoms. Finally, regular contact with a multidisciplinary team may enhance self-efficacy, provide social validation and improve navigation of health and social care systems. These mechanisms may act in combination and could partly explain why participants who reported completion of rehabilitation also reported better Vitality, social functioning and perceived general health.

Our findings are broadly consistent with emerging interventional and systematic evidence on rehabilitation in adults with post-COVID-19 condition. A large systematic review and meta-analysis of rehabilitation interventions for PCC reported that structured programmes lead to improvements in physical capacity (e.g., 6 min walk distance), dyspnoea and health-related quality of life compared with usual care [39]. Randomised controlled trials of supervised, multicomponent exercise-based rehabilitation (RECOVE trial) and cardiopulmonary rehabilitation in long-COVID cohorts likewise demonstrated clinically meaningful gains in exercise tolerance, symptom burden and patient-reported health status [40]. Furthermore, a recent living systematic review of long-COVID interventions concluded that combined physical and mental-health rehabilitation programmes probably improve overall health status, functional outcomes and quality of life when compared with usual care [12]. In contrast to these protocolised trials with clearly defined interventions and eligibility criteria, our study reflects a heterogeneous, real-world mix of rehabilitation modalities accessed by a self-selected group of patients with varying symptom duration and severity. The observation that participants who reported completing rehabilitation had higher Vitality and social functioning scores, better general and mental health and lower functional limitation is therefore in line with the direction of effects seen in controlled settings and suggests that some of the benefits of rehabilitation may extend into routine practice. These observations may help to inform the design and targeting of future prospective rehabilitation programmes for post-COVID-19 condition, by highlighting which patient-reported domains (such as vitality, social functioning and perceived health) are most likely to improve and which subgroups may merit particular attention in controlled studies.

## 5. Strengths and Limitations

This study offers several important strengths. It addresses a clinically relevant and still evolving problem by analysing a relatively large sample of adults with long COVID recruited from two established online support communities. We used well-validated instruments and examined both health-related quality of life and functional status, allowing a nuanced description of how long COVID affects multiple aspects of everyday life. By comparing respondents who did and did not report rehabilitation and by applying multivariable models with conservative Bonferroni correction, we were able to explore how rehabilitation status, demographic factors and symptom duration relate to multiple outcome domains within a real-world population of individuals with ongoing symptoms. Together, these features provide a detailed cross-sectional snapshot of long COVID that complements emerging evidence from clinical cohorts and trials and may help to generate hypotheses for future interventional studies.

Several important limitations must also be acknowledged. First, the cross-sectional design with self-reported exposure and outcomes precludes causal inference about the impact of rehabilitation. Respondents who completed rehabilitation may differ systematically from those who did not with respect to symptom severity, motivation, resources, health literacy or access to care; these and other unmeasured factors could contribute to the observed differences. Second, recruitment via Facebook support groups implies substantial self-selection. The sample may over-represent individuals with more severe or persistent symptoms, those more engaged with their health, or those with higher digital literacy, limiting generalisability to the broader population of people with long COVID. Moreover, because our inclusion threshold was ≥4 weeks of persistent symptoms, the sample combines individuals in post-acute (4–12 weeks) and longer-term phases, so direct comparisons with cohorts defined strictly by the ≥12-week PCC criterion should be made with caution. No additional recruitment channels (e.g., outpatient clinics or population-based sampling frames) were used, and it is therefore likely that our sample over-represents individuals who are more informed about their condition and more motivated to seek rehabilitation than the wider population of people with post-COVID-19 condition.

Third, all data were obtained through online questionnaires without objective clinical assessment, and self-reported anthropometric measures and comorbidities may be imprecise. We were unable to verify the type, intensity, duration or quality of rehabilitation received, nor to assess adherence, and our binary rehabilitation variable likely obscures important heterogeneity in programmes; this is a major limitation and means that ‘rehabilitation’ in our analyses should be interpreted only as a crude proxy for a broad mix of real-world interventions. In addition, we treated the ordinal PCFS grades (0–4) as approximately continuous in the regression analyses, which is a pragmatic modelling simplification and may not fully capture non-linear relationships in functional status; accordingly, the regression results for PCFS should be interpreted with particular caution. Fourth, we did not have pre-COVID or pre-rehabilitation baseline SF-36 or PCFS scores, so we cannot determine whether the observed differences reflect change over time or pre-existing differences between groups. The sample was drawn from two online support communities and does not constitute a probability sample of the general population. The sample size calculation reported in the Methods should therefore be viewed as a conventional benchmark rather than a true estimate of sampling error, and confidence intervals cannot be interpreted as population-representative for any specific country. Finally, although we applied Bonferroni correction to reduce the risk of false-positive findings, this conservative approach increases the risk of type II error and may have masked smaller but clinically relevant associations, particularly in mental-health-related outcomes. These limitations are typical of survey-based research in rapidly developing clinical areas and underscore the need for complementary prospective and experimental designs.

## 6. Implications and Future Directions

Taken together, the findings reinforce the substantial burden of long COVID on quality of life and everyday functioning and add to the limited real-world evidence on how outcomes vary within symptomatic populations. Within the constraints of a cross-sectional design, we observed that younger age, shorter symptom duration and reported completion of rehabilitation were consistently associated with more favourable outcomes across several domains. Interpreted with appropriate caution, these associations are aligned with current recommendations to offer multidisciplinary, person-centred rehabilitation to individuals with long COVID, with careful monitoring of safety—especially in those with post-exertional symptom exacerbation—and tailoring of interventions to individual needs and capacities.

Future research should prioritise prospective cohort studies and randomised or well-controlled pragmatic trials that compare clearly specified rehabilitation programmes with appropriate comparators, using harmonised outcome sets that include SF-36 domains, PCFS and condition-specific measures. Detailed reporting of rehabilitation content, intensity, duration and adherence, together with richer information on comorbidities, socioeconomic circumstances and psychosocial factors, will be essential to clarify which components are most beneficial for which patients and at what stage of recovery. Such work may help to bridge the gap between efficacy signals from controlled trials and the heterogeneous reality of routine rehabilitation services, and to inform the development of scalable, equitable and evidence-based pathways for people living with long COVID. Crucially, because of the cross-sectional design and the absence of pre-COVID or pre-rehabilitation baseline data, these associations cannot be interpreted as evidence that rehabilitation caused the observed differences; longitudinal and controlled studies are required to determine causal effects.

## 7. Conclusions

This cross-sectional online survey of 406 adults with post-COVID-19 condition, recruited from two national online support communities, confirms the substantial and multidimensional burden of long COVID on health-related quality of life and everyday functioning. Across most SF-36 domains and both component summaries, as well as on the PCFS scale, respondents reported marked impairments—particularly in vitality, mental health and role limitations—underscoring the extent to which persistent post-COVID symptoms restrict daily activities and social participation.

Within this symptomatic population, reported completion of rehabilitation was consistently associated with better outcomes. Individuals who had undergone rehabilitation described higher vitality, better mental health, more favourable general and perceived health, and lower functional limitation on the PCFS, and a number of these associations remained significant after conservative Bonferroni correction and adjustment for age, sex and symptom duration. At the same time, older age and longer symptom duration were robust correlates of poorer physical and mental health outcomes, highlighting groups that may require particular attention in clinical practice.

These findings should be interpreted cautiously. The cross-sectional design, self-reported exposure and outcomes, non-probability sampling from Facebook support communities and crude, binary capture of heterogeneous rehabilitation programmes preclude any causal inference and limit generalisability. Nevertheless, the results complement emerging evidence from clinical cohorts and trials by suggesting that, in real-world settings, engagement with rehabilitation is positively associated with multiple aspects of patient-reported health. They support the rationale for integrating accessible, multidisciplinary rehabilitation into long COVID care pathways and highlight the need for prospective, well-controlled studies to determine which rehabilitation components are most effective, for whom and at what stage of recovery.

## Figures and Tables

**Table 1:** Sample characteristics (*n* = 406).

Variable	Category/Summary	*n*	%
Sex	Female	308	75.86
	Male	98	24.14
Age, years	Mean ± SD	36.01 ± 12.09	—
Number of prior COVID-19 infections	1×	176	43.35
	2×	161	39.66
	3×	40	9.85
	≥4×	29	7.14
Persistent symptoms	Yes	406	100.00
Duration of persistent symptoms	>1 year	187	46.06
	7–12 months	80	19.70
	4–6 months	60	14.78
	1–3 months	79	19.46
Rehabilitation completed	No	224	55.17
	Yes	182	44.83

**Table 2:** Baseline characteristics by rehabilitation status: homogeneity tests between respondents who completed rehabilitation (*n* = 182) and those who did not (*n* = 224).

Variable	Rehabilitation: Yes	Rehabilitation: No	Test	Statistic	*p*-Value
Gender	Female: 135 (74.2%) Male: 47 (25.8%)	Female: 173 (77.2%) Male: 51 (22.8%)	χ^2^	χ^2^(1) = 0.36	*p* = 0.549
Age (years)	35.5 ± 11.7 (*n* = 182)	36.4 ± 12.4 (*n* = 224)	Welch *t*-test	t = −0.767 (df ≈ 396)	*p* = 0.443
Height (cm)	171.0 [165.0–179.8] (*n* = 182)	170.0 [164.0–178.0] (*n* = 224)	Mann—Whitney U	U = 18,444.0; Z = −1.651	*p* = 0.099
Weight (kg)	70.0 [60.0–84.0] (*n* = 182)	68.0 [57.8–81.0] (*n* = 224)	Mann—Whitney U	U = 18,989.5; Z = −1.186	*p* = 0.236
BMI (kg/m^2^)	24.1 [21.0–26.7] (*n* = 182)	23.8 [20.7–27.2] (*n* = 224)	Mann—Whitney U	U = 19,685.0; Z = −0.594	*p* = 0.552
Number of COVID-19 infections	2 [1–2] (*n* = 182)	2 [1–2] (*n* = 224)	Mann—Whitney U	U = 20,229.0; Z = −0.142	*p* = 0.887
Persistent symptoms (Yes = 1)	*n* = 182 (100.0%)	*n* = 224 (100.0%)	no between-group test performed		N/A
Symptom duration (1–4)	3 [2–4] (*n* = 182)	3 [2–4] (*n* = 224)	Mann—Whitney U	U = 20,247.5; Z = −0.123	*p* = 0.902
Hospitalisation for COVID-19 (Yes = 1)	20/182 (11.0%)	14/224 (6.2%)	χ^2^	χ^2^(1) = 2.939	*p* = 0.086

Note: Continuous and ordinal variables are presented as mean ± SD or median [IQR], as appropriate. Group comparisons were performed using Welch’s *t*-test for age, Mann–Whitney U tests for height, weight, BMI, number of infections and symptom duration, and χ^2^ tests for categorical variables (gender, hospitalisation). Persistent symptoms were present in 100% of respondents in both groups; no between-group test was performed for this variable.

**Table 3:** Comparison of SF-36 quality of life domains and Post-COVID Functional Status Scale (PCFS) between individuals who completed (*n* = 182) and did not complete (*n* = 224) rehabilitation (Mann–Whitney test).

Health Indicator	Mann—Whitney U	*p*-Value	Effect Size (Rank-Biserial Correlation, r)
Physical Functioning (PF)	18,103.500	0.052	0.112
Role Limitations Due to Physical Health (RP)	16,035.500	<0.001	0.213
Bodily Pain (BP)	14,575.500	<0.001	0.285
General Health (GH)	13,271.000	<0.001	0.349
Vitality (VT)	5237.000	<0.001	0.743
Social Functioning (SF)	13,312.500	<0.001	0.347
Role Limitations Due to Emotional Problems (RE)	14,518.000	<0.001	0.288
Mental Health (MH)	8730.500	<0.001	0.572
Physical Component Summary (PCS)	13,956.500	<0.001	0.315
Mental Component Summary (MCS)	95,27.500	<0.001	0.533
Perceived Health Status (SF-36 single item)	11,692.000	<0.001	0.426
Post-COVID Functional Status (PCFS)	26,246.500	<0.001	0.288

SF-36 = 36-Item Short Form Health Survey; PF = Physical Functioning; RP = Role Limitations due to Physical Health; BP = Bodily Pain; GH = General Health; VT = Vitality; SF = Social Functioning; RE = Role Limitations due to Emotional Problems; MH = Mental Health; PCS = Physical Component Summary; MCS = Mental Component Summary. Perceived Health Status = SF-36 self-rated health item (“In general, would you say your health is…?”); higher scores indicate better perceived overall health. PCFS = Post-COVID Functional Status Scale; higher scores indicate greater functional limitation. For PCFS, the sign of the rank-biserial correlation (r) was reversed so that positive values consistently indicate better outcomes (less functional limitation) in the rehabilitation group compared with the non-rehabilitation group. Positive values of r indicate better outcomes in the rehabilitation group; negative values (not present in this table) would indicate worse outcomes in the rehabilitation group. Note: Comparison was conducted using the Mann–Whitney U test between individuals who completed rehabilitation (*n* = 182) and those who did not (*n* = 224).

**Table 4:** Significant predictors of SF-36 quality of life domains, summary scores, perceived health, and Post-COVID Functional Status Scale (PCFS) in the full sample (*n* = 406) (multiple linear regression; standardised coefficients).

Dependent Variable	Predictor(s)	Standardised β	*p*-Value
Physical Functioning (PF)	Age; Sex; Symptom duration; Rehabilitation	−0.189; −0.182; −0.183; 0.145	<0.001; <0.001; <0.001; 0.002
Role Limitations due to Physical Health (RP)	Age; Number of past COVID-19 infections; Symptom duration; Rehabilitation	−0.130; −0.092; −0.185; 0.202	0.019; 0.049; <0.001; <0.001
Bodily Pain (BP)	Age; Sex; Symptom duration; Rehabilitation	−0.165; −0.134; −0.174; 0.274	0.002; 0.004; <0.001; <0.001
General Health (GH)	Age; Sex; Symptom duration; Rehabilitation	−0.191; −0.107; −0.173; 0.285	<0.001; 0.021; <0.001; <0.001
Vitality (VT)	Symptom duration; Rehabilitation	−0.162; 0.645	<0.001; <0.001
Social Functioning (SF)	Age; Symptom duration; Rehabilitation	−0.218; −0.217; 0.302	<0.001; <0.001; <0.001
Role Limitations due to Emotional Problems (RE)	Age; Physically demanding job; Symptom duration; Rehabilitation	−0.162; 0.100; −0.125; 0.261	0.004; 0.035; 0.020; <0.001
Mental Health (MH)	Age; Symptom duration; Rehabilitation	−0.151; −0.138; 0.486	0.003; 0.005; <0.001
Physical Component Summary (PCS)	Age; Sex; Number of past COVID-19 infections; Symptom duration; Rehabilitation	−0.203; −0.152; −0.094; −0.223; 0.285	<0.001; <0.001; 0.029; <0.001; <0.001
Mental Component Summary (MCS)	Age; Symptom duration; Rehabilitation	−0.183; −0.188; 0.475	<0.001; <0.001; <0.001
Perceived Health Status (SF-36 single item)	Age; Sex; Symptom duration; Rehabilitation	−0.204; −0.104; −0.217; 0.406	<0.001; 0.014; <0.001; <0.001
Post-COVID Functional Status (PCFS)	Rehabilitation	−0.246	<0.001

SF-36 = 36-Item Short Form Health Survey; PCS = Physical Component Summary; MCS = Mental Component Summary; MH = Mental Health; SF = Social Functioning; BP = Bodily Pain; VT = Vitality; GH = General Health; RP = Role Limitations due to Physical Health; RE = Role Limitations due to Emotional Problems; PF = Physical Functioning. Perceived Health Status = SF-36 self-rated health item (“In general, would you say your health is…?”); higher scores indicate better perceived overall health. Post-COVID Functional Status (PCFS) = Post-COVID Functional Status Scale; higher scores indicate greater functional limitation.

**Table 5:** Standardised regression coefficients (β), raw *p*-values and Bonferroni-adjusted *p*-values (*p*_adj) for prespecified predictor–outcome pairs in the multivariable regression analyses (*n* = 406).

Dependent Variable	Predictor	Standardised β	*p* (Uncorrected)	Bonferroni-Adjusted *p* (*p*_adj)
Physical Functioning (PF)	Age	−0.189	<0.001	<0.037
	Sex	−0.182	<0.001	<0.037
	Symptom duration	−0.183	<0.001	<0.037
	Rehabilitation	0.145	0.002	0.074
Role Limitations due to Physical Health (RP)	Age	−0.130	0.019	0.703
	Number of past COVID-19 infections	−0.092	0.049	1.000
	Symptom duration	−0.185	<0.001	<0.037
	Rehabilitation	0.202	<0.001	<0.037
Bodily Pain (BP)	Age	−0.165	0.002	0.074
	Sex	−0.134	0.004	0.148
	Symptom duration	−0.174	<0.001	<0.037
	Rehabilitation	0.274	<0.001	<0.037
General Health (GH)	Age	−0.191	<0.001	<0.037
	Sex	−0.107	0.021	0.777
	Symptom duration	−0.173	<0.001	<0.037
	Rehabilitation	0.285	<0.001	<0.037
Vitality (VT)	Symptom duration	−0.162	<0.001	<0.037
	Rehabilitation	0.645	<0.001	<0.037
Social Functioning (SF)	Age	−0.218	<0.001	<0.037
	Symptom duration	−0.217	<0.001	<0.037
	Rehabilitation	0.302	<0.001	<0.037
Role Limitations due to Emotional Problems (RE)	Age	−0.162	0.004	0.148
	Physically demanding job	0.100	0.035	1.000
	Symptom duration	−0.125	0.020	0.740
	Rehabilitation	0.261	<0.001	<0.037
Mental Health (MH)	Age	−0.151	0.003	0.111
	Symptom duration	−0.138	0.005	0.185
	Rehabilitation	0.486	<0.001	<0.037
Physical Component Summary (PCS)	Age	−0.203	<0.001	<0.037
	Sex	−0.152	<0.001	<0.037
	Number of past COVID-19 infections	−0.094	0.029	1.000
	Symptom duration	−0.223	<0.001	<0.037
	Rehabilitation	0.285	<0.001	<0.037
Mental Component Summary (MCS)	Age	−0.183	<0.001	<0.037
	Symptom duration	−0.188	<0.001	<0.037
	Rehabilitation	0.475	<0.001	<0.037
Perceived Health Status (SF-36 single item)	Age	−0.204	<0.001	<0.037
	Sex	−0.104	0.014	0.518
	Symptom duration	−0.217	<0.001	<0.037
	Rehabilitation	0.406	<0.001	<0.037
Post-COVID Functional Status (PCFS)	Rehabilitation	−0.246	<0.001	<0.037

## Data Availability

The original contributions presented in this study are included in the article/Supplementary Materials. Further inquiries can be directed to the corresponding author.

## Supplementary Materials

The following supporting information can be downloaded: https://www.mdpi.com/article/10.3390/medicina61122214/s1. Supplementary File S1: Study questionnaire used in the survey, including demographic and clinical questions, long COVID-related items, and questions on rehabilitation and perceived impact of rehabilitation. Only study-specific items are included. Standardised instruments (the SF-36 Health Survey and the Post-COVID Functional Status [PCFS] Scale) are not reproduced due to copyright restrictions and can be obtained from their original sources as cited in the main manuscript.

## References

[1] Luo D., Mei B., Wang P., Li X., Chen X., Wei G., Kuang F., Li B., Su S. (2023). Prevalence and Risk Factors for Persistent Symptoms after COVID-19: A Systematic Review and Meta-Analysis. Clin. Microbiol. Infect..

[2] Fernandez-de-las-Peñas C., Notarte K.I., Macasaet R., Velasco J.V., Catahay J.A., Ver A.T., Chung W., Valera-Calero J.A., Navarro-Santana M. (2024). Persistence of Post-COVID Symptoms in the General Population Two Years after SARS-CoV-2 Infection: A Systematic Review and Meta-Analysis. J. Infect..

[3] Davis H., McCorkell L., Vogel J.M., Topol E.J. (2023). Long COVID: Major Findings, Mechanisms and Recommendations. Nat. Rev. Microbiol..

[4] Bonsaksen T., Leung J., Price D., Ruffolo M., Lamph G., Kabelenga I., Thygesen H., Geirdal A.Ø. (2022). Self-Reported Long COVID in the General Population: Sociodemographic and Health Correlates in a Cross-National Sample. Life.

[5] Davis H., Assaf G., McCorkell L., Wei H., Low R., Re’em Y., Redfield S., Austin J.P., Akrami A. (2021). Characterizing Long COVID in an International Cohort: 7 Months of Symptoms and Their Impact. eClinicalMedicine.

[6] Sykes D.L., Holdsworth L., Jawad N., Gunasekera P., Morice A.H., Crooks M.G. (2021). Post-COVID-19 Symptom Burden: What Is Long-COVID and How Should We Manage It?. Lung.

[7] Todt B.C., Szlejf C., Duim E., Linhares A.O.M., Kogiso D., Varela G., Campos B.A., Fonseca C.M.B., Polesso L.E., Bordon I.N.S. (2021). Clinical Outcomes and Quality of Life of COVID-19 Survivors: A Follow-up of 3 Months Post Hospital Discharge. Respir. Med..

[8] Oronsky B., Larson C., Hammond T., Oronsky A.L., Kesari S., Lybeck M., Reid T.R. (2021). A Review of Persistent Post-COVID Syndrome (PPCS). Clin. Rev. Allergy Immunol..

[9] Sun C., Liu Z., Li S., Wang Y., Liu G. (2024). Impact of Long COVID on Health-Related Quality of Life Among Patients After Acute COVID-19 Infection: A Cross-Sectional Study. Inq. J. Health Care Organ. Provis. Financ..

[10] Leite L.C., de Carvalho L.R., de Queiroz D.M., Farias M., Cavalheri V., Edgar D.W., Nery B.R., Barros N.V., Maldaner V., Campos N.G. (2022). Can the Post-COVID-19 Functional Status Scale Discriminate between Patients with Different Levels of Fatigue, Quality of Life and Functional Performance?. Pulmonology.

[11] Schild A., Scharfenberg D., Kirchner L., Klein K., Regorius A., Goereci Y., Meiberth D., Sannemann L., Lülling J., Schweitzer F. (2023). Subjective and Objective Cognitive Deficits in Patients with Post-COVID Syndrome. Z. Neuropsychol..

[12] Zeraatkar D., Ling K., Kirsh S., Jassal T., Shahab M., Movahed H., Talukdar J.R., Walch A., Chakraborty S., Turner T. (2024). Interventions for the Management of Long Covid (Post-Covid Condition): Living Systematic Review. BMJ.

[13] Chuang H., Lin C.-W., Hsiao M., Wang T., Liang H. (2023). Long COVID and Rehabilitation. J. Formos. Med. Assoc..

[14] D’Souza A.F. (2021). Long COVID: Implications for Physiotherapy. Eur. J. Physiother..

[15] Ostrowska M., Rzepka-Cholasińska A., Pietrzykowski Ł., Michalski P., Kosobucka A., Jasiewicz M., Kasprzak M., Kryś J., Kubica A. (2023). Effects of Multidisciplinary Rehabilitation Program in Patients with Long COVID-19: Post-COVID-19 Rehabilitation (PCR SIRIO 8) Study. J. Clin. Med..

[16] Palermi S., Compagno S., Pescatore V., Brugin E., Tegon G., Sarto M., Marin R., Calzavara V., Nizzetto M., Scevola M. (2022). Physical and Psychological Reconditioning in Long Covid Syndrome Patients: Results of a Structured Physical Exercise Program. Eur. J. Prev. Cardiol..

[17] Zheng C., Chen X.-K., Sit C.H.P., Liang X., Li M., Chun-Hang A., Wong S.H. (2023). Effect of Physical Exercise–Based Rehabilitation on Long COVID: A Systematic Review and Meta-Analysis. Med. Sci. Sports Exerc..

[18] Romanet C., Wormser J., Cachanado M., Santiago M.G., Châtellier G., Valenza M.C., Philippart F. (2024). Effectiveness of Physiotherapy Modalities on Persisting Dyspnoea in Long COVID: A Systematic Review and Meta-Analysis. Respir. Med..

[19] Ewing A.G., Joffe D., Blitshteyn S., Brooks A.E.S., Wist J., Yam Y.B., Bilodeau S., Curtin J., Duncan R., Faghy M.A. (2024). Long COVID Clinical Evaluation, Research and Impact on Society: A Global Expert Consensus. Ann. Clin. Microbiol. Antimicrob..

[20] Moreels S., Bensemmane S., Schreye R.D., Cuschieri S. (2024). Caring for Long Covid Patients in Primary Healthcare: A Cross-Sectional Study on General Practitioners’ Knowledge, Perception and Experience in Belgium and Malta. BMC Prim. Care.

[21] Gamillscheg P., Łaszewska A., Kirchner S., Hoffmann K., Simon J., Mayer S. (2024). Barriers and Facilitators of Healthcare Access for Long COVID-19 Patients in a Universal Healthcare System: Qualitative Evidence from Austria. Int. J. Equity Health.

[22] Nerli T.F., Selvakumar J., Cvejic E., Heier I., Pedersen M., Johnsen T.L., Wyller V.B. (2024). Brief Outpatient Rehabilitation Program for Post–COVID-19 Condition. JAMA Netw. Open.

[23] León-Herrera S., Oliván-Blázquez B., Sánchez-Recio R., Méndez-López F., Botaya R.M., Sánchez-Arizcuren R. (2024). Effectiveness of an Online Multimodal Rehabilitation Program in Long COVID Patients: A Randomized Clinical Trial. Arch. Public Health.

[24] Gartmann J., Sturm C., Boekel A. (2024). Physiotherapy Interventions in Post- and Long-COVID-19: A Scoping Review Protocol. BMJ Open.

[25] World Health Organization. Regional Office for Europe (2025). Impact of the COVID-19 Pandemic on the Rehabilitation Workforce and Service Delivery in Five Countries of the WHO European Region: Armenia, Georgia, Italy, Poland and the United Kingdom.

[26] Manderson K., Taylor N.F., Lewis A.K., Harding K.E. (2025). Service-Level Interventions to Reduce Waiting Time in Outpatient and Community Health Settings May Be Sustained: A Systematic Review. BMJ Open Qual..

[27] Carlile O., Briggs A., Henderson A.D., Butler-Cole B.F.C., Tazare J., Tomlinson L.A., Marks M., Jit M., Lin L.-Y., Bates C. (2024). Impact of Long COVID on Health-Related Quality-of-Life: An OpenSAFELY Population Cohort Study Using Patient-Reported Outcome Measures (OpenPROMPT). Lancet Reg. Health–Eur..

[28] Klok F.A., Boon G.J.A.M., Barco S., Endres M., Geelhoed J.J.M., Knauß S., Rezek S., Spruit M.A., Vehreschild J.J., Siegerink B. (2020). The Post-COVID-19 Functional Status Scale: A Tool to Measure Functional Status over Time after COVID-19. Eur. Respir. J..

[29] Ware J.E., Sherbourne C.D. (1992). The MOS 36-Item Short-Form Health Survey (SF-36): I. Conceptual Framework and Item Selection. Med. Care.

[30] Skalská H., Sobotík Z., Jezberová D., Mares J. (2000). Use and evaluation of the Czech version of the SF-36 questionnaire self-reported health status of medical students. Cent. Eur. J. Public Health.

[31] Greenhalgh T., Sivan M., Perlowski A., Nikolich-Žugich J. (2024). Long COVID: A Clinical Update. Lancet.

[32] Líška D., Liptáková E., Babičová A., Baťalík L., Baňárová P.S., Dobrodenková S. (2022). What Is the Quality of Life in Patients with Long COVID Compared to a Healthy Control Group?. Front. Public Health.

[33] Asimakos A., Spetsioti S., Mavronasou A., Gounopoulos P., Siousioura D., Dima E., Gianniou N., Sigala I., Zakynthinos G.Ε., Kotanidou A. (2023). Additive Benefit of Rehabilitation on Physical Status, Symptoms and Mental Health after Hospitalisation for Severe COVID-19 Pneumonia. BMJ Open Respir. Res..

[34] Goërtz Y.M.J., Herck M.V., Delbressine J.M., Vaes A.W., Meys R., Machado F., Houben-Wilke S., Burtin C., Posthuma R., Franssen F.M.E. (2020). Persistent Symptoms 3 Months after a SARS-CoV-2 Infection: The Post-COVID-19 Syndrome?. ERJ Open Res..

[35] Halpin S., McIvor C., Whyatt G., Adams A., Harvey O.J., McLean L., Walshaw C., Kemp S.A., Corrado J., Singh R. (2020). Postdischarge Symptoms and Rehabilitation Needs in Survivors of COVID-19 Infection: A Cross-sectional Evaluation. J. Med. Virol..

[36] Samuelsson C.M., Hussain N., Drummond A., Persson C.U. (2025). Health-Related Quality of Life One Year After Intensive Care Unit Admission for COVID-19: A Retrospective, Cross-Sectional, Longitudinal Observational Study. Health Sci. Rep..

[37] DeMars J., Brown D.A., Angelidis I., Jones F., McGuire F.A., O’Brien K.K., Oller D., Pemberton S., Tarrant R., Verduzco-Gutierrez M. (2022). What Is Safe Long COVID Rehabilitation?. J. Occup. Rehabil..

[38] Samper-Pardo M., León-Herrera S., Oliván-Blázquez B., Gascón S., Sánchez-Recio R. (2023). Clinical Characterization and Factors Associated with Quality of Life in Long COVID Patients: Secondary Data Analysis from a Randomized Clinical Trial. PLoS ONE.

[39] Pouliopoulou D.V., Macdermid J.C., Saunders E., Peters S., Brunton L., Miller E., Quinn K.L., Pereira T.V., Bobos P. (2023). Rehabilitation Interventions for Physical Capacity and Quality of Life in Adults With Post-COVID-19 Condition: A Systematic Review and Meta-Analysis. JAMA Netw. Open.

[40] Jimeno-Almazán A., Buendía-Romero Á., Martínez-Cava A., Franco-López F., Sánchez-Alcaraz B.J., Courel-Ibáñez J., Pallarés J.G. (2023). Effects of a Concurrent Training, Respiratory Muscle Exercise, and Self-Management Recommendations on Recovery From Post-COVID-19 Conditions: The RECOVE Trial. J. Appl. Physiol..

