# Supplementary material for: Quality of Life and Functional Status in Individuals with Persistent Post-COVID Symptoms: A Cross-Sectional Comparison by Reported Rehabilitation
Source: Medicina (Kaunas). 2025 Dec 15;61(12):2214. doi: 10.3390/medicina61122214 (PMC12734895; doi:10.3390/medicina61122214)
Supplement: Supplementary file 1 [file medicina-61-02214-s001.zip › medicina-4021885-supplementary.pdf]

Supplementary File S1. Study Questionnaire (selected items, without standardized instruments)

Quality of life after overcoming the disease COVID-19

Hello, my name is Michal Macej and I am a doctoral student and student of Public Health at the Faculty of Medicine of the UPJŠ in Košice, Slovakia. In my dissertation, I deal with patients after overcoming the disease of COVID-19 and the impact of this disease on their quality of life. I would be grateful if you could take a few minutes to complete this online research questionnaire. The survey is anonymous.

* Marks a mandatory question

Sex *
Select only one option

- Male
- Female

Age *

[open numeric field]

Height (cm) *

[open numeric field]

Weight (kg) *

[open numeric field]

I have a physically demanding job *
Select only one option

- Yes
- No

Before my COVID-19 disease, I was physically active and regularly performed some form of physical or sports activity *
Select only one option

- Yes
- No

In the past, I overcame the disease of COVID-19 *
Select only one option

- one time
- two times
- three times
- four times or more

Date of overcoming the last illness of COVID-19 (month, year) *

[open field: month/year]

I have/had persistent and long-term difficulties even after overcoming the disease (so-called long COVID) *
Select only one option

- Yes
- No

If so, how long have these difficulties persisted?
Select only one option

- 1–3 months
- 3–6 months
- 6–12 months
- more than a year

These persistent symptoms include:
Check all valid options

- Fatigue
- Problems with memory and concentration
- Aggravation after physical activity

- Headache
- Sleep problems
- Palpitations
- Loss of taste
- Joint pain
- Muscle pain
- Anxiety
- Breathing difficulties
- Persistent cough
- Loss of smell
- Chest pain
- Depression
- Disorder of balance and gait
- Dizziness
- Diarrhoea
- Stomach pain
- Skin problems
- Numbness and tingling in the limbs
- Changes in the menstrual cycle
- A sore throat
- Cramps
- Tinnitus
- Tremor
- Psychological problems
- **Other:** [open field]

I was hospitalized with COVID-19 *
Select only one option

- Yes
- No

Compared to your pre-COVID-19 condition, how would you rate your health in general now? *
Select only one option

- much better
- somewhat better
- about the same
- somewhat worse
- much worse

In the past, I underwent rehabilitation treatment in order to mitigate the impact of this disease *
Select only one option

- Yes
- No

If so, was it:
Select only one option

- Outpatient form
- Spa treatment
- Hospital rehabilitation
- Rehabilitation in a social services home
- Private form of rehabilitation
- **Others:** [open field]

If not, why?
Check all valid options

- Rehabilitation was not necessary due to my health condition
- Lack of time
- I was not offered this option
- I was not interested
- I didn't know about such a possibility
- Long distance
- Transport problems
- **Others:** [open field]

If you completed rehabilitation treatment, how much did it affect your difficulties after COVID?
Select only one option

- did not affect at all
- slightly improved
- improved to a great extent
- completely improved
- made my condition worse

The participant of this anonymous survey confirms that they participated voluntarily and also express their consent to the processing of their results for the purposes of

scientific publication. Completing this response is mandatory.
Select only one option

- Yes, I confirm
- No, I do not confirm

Note on standardized instruments (not included in this file)

In this study, we additionally used two standardized instruments:

- the 36-Item Short Form Health Survey (SF-36) to assess health-related quality of life, and
- the Post-COVID Functional Status (PCFS) Scale to assess functional status after COVID-19.

Due to copyright restrictions, the **full item content of the SF-36 and the PCFS Scale is not reproduced in this supplementary file**. Only study-specific (demographic and clinical) questions are included here. The standardized questionnaires can be obtained from their original sources and are cited in the main manuscript.

Standardized instruments (SF-36 and PCFS) are therefore used and reported in accordance with their licensing and copyright conditions, but their full wording is not disclosed in the supplementary materials.
